# Protective Effects of Grape Seed Proanthocyanidins on the Kidneys of Diabetic Rats through the Nrf2 Signalling Pathway

**DOI:** 10.1155/2020/5205903

**Published:** 2020-09-29

**Authors:** Yusong Ding, Haiyan Li, Yang Li, Dandan Liu, Liyuan Zhang, Tongling Wang, Tao Liu, Long Ma

**Affiliations:** ^1^College of Public Health, Xinjiang Medical University, Urumqi 830011, China; ^2^Department of Public Health, Shihezi University School of Medicine, Shihezi, Xinjiang 832000, China

## Abstract

**Background:**

Diabetic nephropathy (DN) is the most common cause of end-stage renal failure. Grape seed proanthocyanidin extract (GSPE) is a powerful antioxidant that is believed to protect the kidney through antioxidant action. However, the underlying mechanism of GSPE protection against DN remains unclear.

**Objective:**

To explore if GSPE can improve DN by activating nuclear factor erythroid 2-related factor 2 (Nrf2) antioxidant response element signalling and to clarify its possible mechanism. *Materials and methods*. Ten healthy Sprague-Dawley rats were randomly selected as controls. Rats with streptozotocin-induced diabetes were randomly divided into three groups (10 animals/group): type 2 diabetes mellitus (T2DM) group (untreated), L-GSPE group (treated with 125-mg/kg/day GSPE for 8 weeks), and H-GSPE group (treated with 250 mg/kg/day GSPE for 8 weeks).

**Results:**

Renal histopathological results indicated limited pathological damage in GSPE-treated groups. Compared with the T2DM group, the H-GSPE group had significantly reduced kidney weight and renal index. Similarly, the levels of fasting blood glucose, serum creatinine, blood urea nitrogen, uric acid, urinary albumin, and renal malondialdehyde (*p* < 0.05) were also significantly decreased. In addition, GSPE significantly increased the levels of superoxide dismutase, total antioxidative capability, and glutathione (*p* < 0.05) as well as the protein levels of Nrf2, HO-1, glutathione S-transferase, and NAD (P)H quinone oxidoreductase 1 (*p* < 0.05).

**Conclusion:**

The results indicate that GSPE reduced renal damage in rats with diabetes by activating the Nrf2 signalling pathway, which consequently increased the antioxidant capacity of the tissue. Therefore, GSPE is a potential natural agent for the treatment of diabetic nephropathy.

## 1. Introduction

Diabetes mellitus is a metabolic syndrome which results from inadequate insulin or impaired action of insulin. According to the report of the International Diabetes Federation [1], about 463 million adults suffer from diabetes globally in 2019. The increase in the incidence of diabetes has led to higher prevalence of complications such as DN [2], and approximately 20–40% of patients with diabetes develop DN [3]. DN is the main cause of end-stage renal disease, accounting for approximately 50% of cases in the developed countries [4]. It is also an independent risk factor for the development and progression of cardiovascular diseases [5], placing a heavy economic burden on healthcare systems globally.

Hyperglycaemia in diabetes can activate the polyol pathway, the hexosamine pathway, the protein kinase C pathway, advanced glycation end products, and the glyceraldehyde autoxidation pathway. These pathways are possibly involved in the production of reactive oxygen species (ROS) and the development of oxidative stress, which are associated with the pathogenesis of diabetes and its complications [6]. Increased production of cellular ROS gradually depletes the antioxidant defence systems, which results in increased oxidative stress. Therefore, upregulating the expression of various endogenous antioxidant genes may become a new strategy for the prevention and treatment of DN. Nrf2 regulates the expression of antioxidant proteins, inhibits the oxidative stress response [7], and plays a key role in the antioxidant defence system. A large number of studies have shown that Nrf2-knockout diabetic mice show more severe kidney damage than wild-type diabetic mice [8, 9]. In addition, the activation of Nrf2 improves DN in mice [10], indicating that Nrf2 plays a key role in the development of DN. Therefore, Nrf2 is a feasible target in the treatment of DN.

Proanthocyanidins are natural antioxidants and complex polymers of flavonoids [11]. Grains, legume seeds, various vegetables, and especially fruits contain high level of proanthocyanidins. In recent years, many studies have reported their various pharmacological activities, such as free radical scavenging, antioxidation, and anti-inflammatory activities. Proanthocyanidins have potential clinical value and application prospects as a therapeutic agent for diabetic comorbidities [12, 13].

Based on the above analysis, we hypothesised that GSPE may regulate the downstream antioxidant enzymes by activating the Nrf2-ARE pathway to inhibit renal oxidative damage and renal dysfunction caused by high glucose and fat in diabetic rats, thereby preventing DN. In the present study, we used an STZ-induced DN rat model to observe the effects of GSPE on STZ-induced DN. In order to ascertain whether a protective effect of GSPE on DN occurred by activating the Nrf2 pathway.

## 2. Materials and Methods

### 2.1. Reagents

Grape seed proanthocyanidins and STZ were obtained from Shanghai Chemical Reagent and Sigma, St. Louis, USA, respectively. Glutathione (GSH) kit (A006-2), superoxide dismutase (SOD) kit (A001-3), the protein standard assay kit (A045-3), total antioxidative capability (T-AOC) kit (A015), and malondialdehyde (MDA) kit (A003-1) were obtained from Nanjing built biological products. Mouse monoclonal antibody (A180), mouse monoclonal antibody (HO-1), goat polyclonal antibody (GST pi), and rabbit polyclonal antibody (ab137550) were obtained from Abbott (Shanghai).

### 2.2. Animals

A total of 48 healthy SPF-grade SD rats (provided by Xinjiang Medical University) with an average body weight of 210 ± 10 g were used for the experiment. The rats were weighed on the first day of the study, and 10 rats were randomly selected for the control group. The remaining 38 rats were fed high-sugar and high-lipid fodder (45% fat, 20% protein, and 35% carbohydrates, as a percentage of total kilocalories) for 4 weeks. The control group was intraperitoneally injected with 1% citric acid buffer solution (pH 4.5). A freshly prepared solution of STZ in 1% citric acid buffer (pH 4.5) was intraperitoneally injected into the mice (40 mg/kg) in the experimental groups (two injections, with a 48 h interval). About 72 h after the second injection of STZ, the rats with fasting blood glucose (FBG) levels >16.7 mmol/L were considered diabetic, and eight rats were excluded for failure to meet this criterion. The diabetic rats were randomly divided into three groups of 10 animals each: T2DM (diabetic rats), L-GSPE, and H-GSPE groups. Different doses were chosen to observe dose-response relationships. The L-GSPE and the H-GSPE groups were administered 125 mg/kg/day and 250 mg/kg/day GSPE by gavage, respectively, for 8 weeks. The control group and the T2DM group were administered normal saline by gavage. During the experiment, the control group was fed basic feed, and the other three treatment groups were fed a high-fat diet [14]. The body weight and blood glucose levels were regularly measured, and their activities were observed and recorded. At the end of the treatment period, urine samples were collected in a metabolic cage within 24 h. Urine was centrifuged at 3,000 rpm for 15 min at 4°C, and the supernatant was stored at −80°C. At the end of the study, the rats were fasted for 18 h, anesthetised with pentobarbital sodium (60 mg/kg, intraperitoneally; Sigma-Aldrich; Merck, Darmstadt, Germany), and sacrificed by cervical decapitation. All the rats were weighed before the sacrifice. Moreover, blood was collected from the heart before sacrifice and centrifuged (4°C, 20 min); the serum was separated and stored at −80°C until use. The kidneys were cut into coronal halves and weighed, and two pieces of kidney were stored at −80°C for western blot analysis. The remaining specimens were fixed in 4% paraformaldehyde buffer at 4°C followed by paraffin treatment and hematoxylin and eosin staining.

### 2.3. Assessment of Biochemical Parameters

The serum and urine were removed from the −80°C freezer and placed at room temperature (22°C) for 30 min, and the levels of serum creatinine (Scr), blood urea nitrogen (BUN), insulin, uric acid (UA), and urinary albumin (UAD) were measured by an automatic biochemical analyser. Homeostasis model assessment-insulin resistance (HOMA-IR) was calculated as (insulin°×°FBG)/22.5. The formula for the renal index is kidney weight (g)/animal weight (g)°×°100.

### 2.4. Measurement of MDA and Antioxidant Enzymes

MDA content, levels of SOD, T-AOC, and GSH in kidney tissue were measured using commercial kits according to the manufacturer's instructions.

### 2.5. Western Blot Analysis

Renal tissues (50 mg) were homogenised in 0.5 mL RIPA buffer at 4°C. The homogenate was centrifuged at 12,000 rpm for 15 min, and protein samples were extracted from the renal tissue at 4°C. Protein concentration was determined with bicinchoninic acid. Equal amounts (40 *μ*g) of protein were loaded and separated by 10% sodium dodecyl sulphate and polyacrylamide gel electrophoresis, and the proteins separated were transferred to nitrocellulose membranes. The membranes were blocked at room temperature for 2 h with 5% skim milk and incubated overnight at 4°C with the primary antibodies GAPDH (1 : 2,000), Nrf2 (1 : 1,000), HO-1 (1 : 1,000), GST (1 : 1,000), and NQO1 (1 : 1,000). The membranes were washed thrice with PBST (TBS and 20% Tween 20) for 5 min each time, incubated with the secondary antibodies at room temperature for 2 h, and washed thrice with PBST. Finally, the membranes were soaked with enhanced chemiluminescence reagent and exposed to X-ray film. Image Lab 4.1 was used to detect and analyse the results. All western blot analyses were performed in triplicate.

### 2.6. RNA Extraction and Quantitative Real-Time Polymerase Chain Reaction (PCR)

Total RNA was extracted from the renal cortex using TRIzol (Invitrogen, Carlsbad, CA, USA) and reverse transcribed into cDNA using the Transcriptor First Strand cDNA Synthesis Kit (Roche, Basel, Switzerland) according to the manufacturer's instructions. The primer sequences for real-time PCR analysis are shown in Table 1. All reactions were conducted in triplicate. The data were analysed using the 2^−ΔΔ*cr*^ method.

### 2.7. Statistical Analysis

Statistical analyses were performed using SPSS and GraphPad Prism software. The data followed a normal distribution and are presented as the mean ± standard deviation. Comparisons between groups were analysed using one-way analysis of variance, and comparisons between pairwise groups were determined with the Bonferroni test. A *p* value < 0.05 indicated statistical significance.

## 3. Results

### 3.1. Effects of GSPE on Body Weight, FBG and Insulin Levels, Homeostasis Model Assessment of Insulin Resistance (HOMA-IR), Kidney Weight, and Renal Index of the Diabetic Rats

As shown in Table 2, FBG level, HOMA-IR, kidney weight, and renal index of diabetic rats were significantly increased but body weight and the level of insulin were significantly decreased compared with those of the control rats (*p* < 0.05). Compared with the diabetic rats, the rats in the L-GSPE and H-GSPE groups had decreased FBG level, HOMA-IR, kidney weight, and renal index but increased body weight (*p* < 0.05).

### 3.2. Effect of GSPE on Renal Pathological Changes in the Diabetic Rats

**Figure 1:**
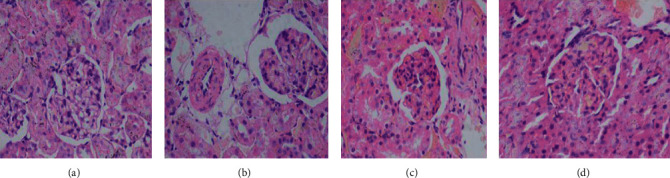
GSPE protects streptozotocin-induced diabetic nephropathy in rats with diabetes: (a) control group; (b) diabetic group; (c) 125 mg/kg GSPE treatment group; (d) 250 mg/kg GSPE treatment group. Original magnification, ×400.

Hematoxylin and eosin staining showed that the glomerular volume of the T2DM group had increased and the basement membrane of the glomerular capillary had thickened. Generally, the width of mesangial hyperplasia was smaller than the capillary diameter, showing a segmental distribution. Renal vascular lesions were obvious, and afferent glomerular arteriole showed hyperplasia and sclerosis. In addition, there was no abnormality in the L-GSPE group except basement membrane thickening. Similarly, no abnormality was seen in the H-GSPE treatment group (Figure 1).

### 3.3. Effects of GSPE on the Renal Function of the Diabetic Rats

**Figure 2:**
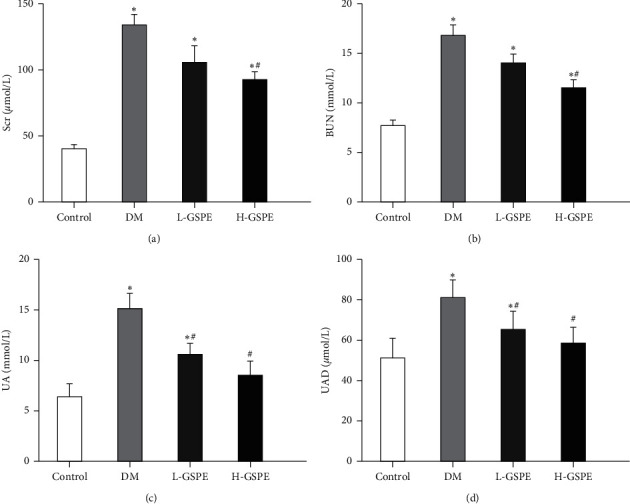
GSPE reduces kidney damage in rats with diabetes. Scr, serum creatinine; BUN, blood urea nitrogen; UA, uric acid; UAD, urinary albumin; Control, normal control group; T2DM, diabetic group; L-GSPE, 125 mg/kg GSPE treatment group; H-GSPE, 250 mg/kg GSPE treatment group. Values are presented as mean ± standard deviation of 10 rats in each group (^*∗*^*p* < 0.05 vs. the control group; ^#^*p* < 0.05 vs. the diabetic group).

The levels of Scr, BUN, UA, and UAD in the T2DM group were significantly increased compared with those in the normal rats. Moreover, compared with the diabetic rats, the rats in the H-GSPE group had decreased levels of Scr, BUN, UA, and UAD (*p* < 0.05, Figure 2). There was no significant deference between the T2DM group and L-GSPE group with regards to the levels of Scr and BUN (*p* < 0.05, Figure 2). However, UA and UAD were significantly decreased in the L-GSPE group (*p* < 0.05, Figure 2).

### 3.4. Effects of GSPE on Renal MDA, SOD, T-AOC, and GSH in the Diabetic Rats

**Figure 3:**
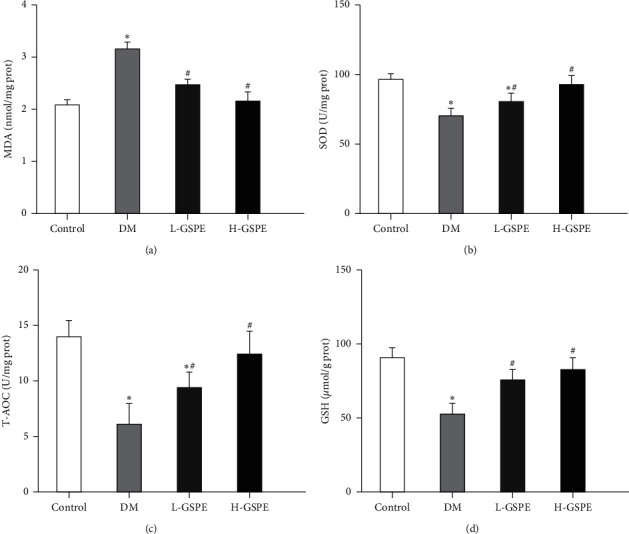
Effects of GSPE on the levels of MDA, SOD, T-AOC, and GSH in diabetic rats. Control, normal control group; T2DM, diabetic group; L-GSPE, 125 mg/kg GSPE treatment group; H-GSPE, 250 mg/kg GSPE treatment group. Values are presented as mean ± standard deviation from 10 rats in each group (^*∗*^*p* < 0.05 vs. the control group; ^#^*p* < 0.05 vs. the diabetic group).

Compared with the normal rats, the levels of SOD, T-AOC, and GSH in the diabetic rats were significantly reduced, whereas the levels of MDA were increased (*p* < 0.05). After treatment with GSPE (250 mg/kg) for 8 weeks, the renal MDA levels significantly decreased in the diabetic rats. Meanwhile, the levels of SOD, T-AOC, and GSH increased (*p* < 0.05, Figure 3).

### 3.5. Effects of GSPE on the Protein Expression of Nrf2, HO-1, GST, and NQO1 in the Diabetic Rats

**Figure 4:**
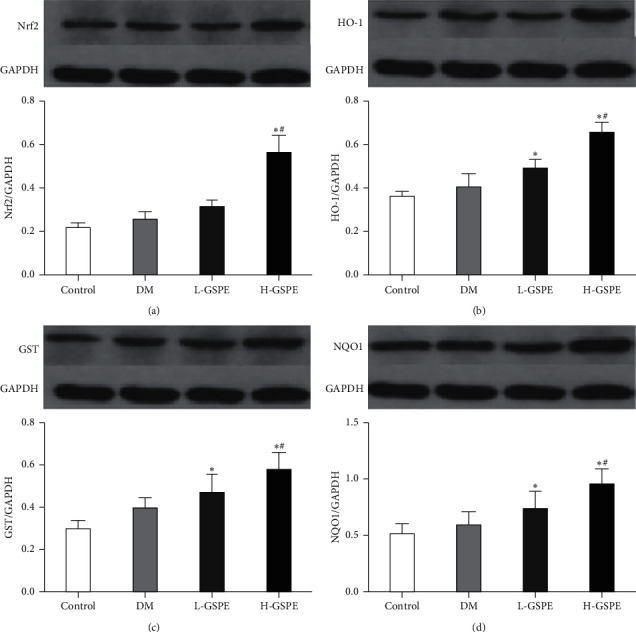
Effects of GSPE on the expression of Nrf2, HO-1, GST, and NQO1. Control, normal control group; T2DM, diabetic group; L-GSPE, 125 mg/kg GSPE treatment group; H-GSPE: 250 mg/kg GSPE treatment group. Values are presented as mean ± standard deviation from 10 rats in each group (^*∗*^*p* < 0.05 vs. the control group; ^#^*p* < 0.05 vs. the diabetic group).

Western blot analysis showed that in the diabetic rats, GSPE treatment (250 mg/kg) significantly upregulated the protein levels of Nrf2. Meanwhile, the protein levels of HO-1, GST, and NQO1 were significantly increased by GSPE treatment (250 mg/kg) (*p* < 0.05, Figure 4). However, compared with diabetic rats, the protein levels of Nrf2, HO-1, GST, and NQO1 were not significantly different in rats of the L-GSPE group (*p* < 0.05, Figure 4).

### 3.6. Nrf2 Promoted the Activation of Nrf2 Signalling in Diabetic Rats

**Figure 5:**
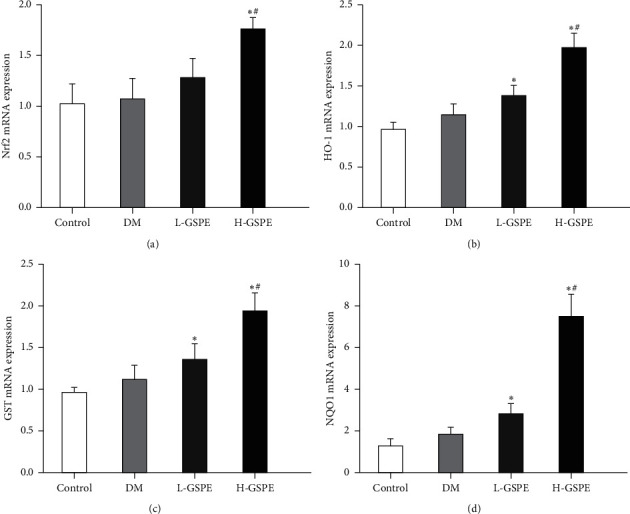
Effects of GSPE on the activation of Nrf2 signalling. Control, normal control group; T2DM, diabetic group; L-GSPE: 125 mg/kg GSPE treatment group; H-GSPE: 250 mg/kg GSPE treatment group. Values are presented as mean ± standard deviation from 10 rats in each group (^*∗*^*p* < 0.05 vs. the control group; #*p* < 0.05 vs. the diabetic group).

The results of real-time PCR showed that the mRNA level of Nrf2 in renal tissues of the H-GSPE group was significantly upregulated compared with those of the diabetic group and control group (*p* < 0.05). The mRNA levels of HO-1, GST, and NQO1 in the renal tissues of the H-GSPE group were significantly upregulated compared with those of the diabetic and control groups (*p* < 0.05, Figure 5).

## 4. Discussion

Currently, different adverse reactions such as hypoglycaemia, digestive tract discomfort, and renal toxicity are associated with the clinical treatment of type 2 diabetes [15, 16]. The development of safe and effective hypoglycaemic drugs is an important task in drug development. Natural products are important targets for drug discovery and research. In this study, diabetic rats treated with GSPE showed upregulated mRNA levels of Nrf2 and downstream genes and increased content of Nrf2 and downstream-related proteins. Furthermore, GSPE can alleviate oxidative damage of the kidney and prevents changes on the renal function and renal pathological change. It also reduced insulin resistance.

These results indicate that GSPE reduced renal damage in T2DM rats by activating the Nrf2 signalling pathway to increase antioxidant capacity. According to the International Diabetes Federation, China has the largest number of patients with diabetes in 2019, reaching 116 million, and the prevalence is expected to reach 140 million by 2030 [1]. DN has become a major public health problem that endangers the health of urban and rural residents of China. It is one of the serious microvascular complications of diabetes, and its onset is not clear as the early symptoms of the diseases are often not obvious. Biochemical indicators and renal pathological conditions often deteriorate when diabetes progresses to DN. Early treatment of diabetes can control the occurrence and development of DN. Because the kidney is more sensitive to oxidative damage, increasing attention is being paid to the treatment of DN through the use of antioxidant strategies [17]. Currently, there are no satisfactory treatments for DN, and hence, it is of great significance to protect the renal function of patients with diabetes and prevent DN.

In the present study, L-GSPE and H-GSPE treatment significantly reduced blood glucose, kidney weight, HOMA-IR, renal index, increased insulin, and body weight of the rats, though the L-GSPE group was slightly worse than the H-GSPE group in reducing HOMA-IR and renal index, but the overall data still indicate that L-GSPE and H-GSPE groups both of them have a significant protective effect against diabetes. The renal index levels and 24-h UAD levels in the H-GSPE group were significantly decreased compared with those in the T2DM group, similar to the study of Gao et al. [14], which explored the protective effect of GSPE through the caspase-12 pathway, but their study had no significant changes in BUN and Scr levels, whereas BUN, Scr, and UA levels were significantly decreased in our study, and this is consistent with the results observed in another study which researched GSPE through its antioxidative activity and anti-inflammatory effects [18]. We also observed that both L-GSPE and H-GSPE groups could reduce UA, UAD, Scr, and BUN, although there was no significant difference in Scr and BUN between L-GSPE and DM groups, indicating that both L-GSPE and H-GSPE groups can reduce the kidney injury caused by diabetes. Histopathological examination of kidney sections of rats showed obvious renal injury, whereas GSPE alleviated renal pathological damage, especially in the H-GSPE group. No abnormalities were found, which is consistent with the findings of Li et al. which researched through mechanism [19], indicating that GSPE can delay the progression of DN in diabetic rats and improve DN-induced kidney damage.

An increase in the level of MDA is a marker of oxidative stress in tissue damage, and it has been demonstrated that SOD and MDA are related to DN [20, 21]. In this study, the level of MDA in kidney tissue homogenates of STZ-induced diabetic rats was significantly increased (*p* < 0.05), suggesting that lipid peroxidation was enhanced. L-GSPE and H-GSPE treatment effectively increased the activity of SOD, T-AOC, and GSH in the kidney tissue of STZ-induced rats and reduced the content of MDA, although the effect of the L-GSPE group is slightly worse than that of the H-GSPE group. This finding may be attributed to the powerful antioxidant activity of GSPE, which has also been demonstrated in previous studies [18, 22]. Therefore, the protective effect of GSPE against diabetic kidney injury may be mediated by its antioxidant effect.

Study has shown that Nrf2 activity is reduced in a condition of oxidative stress, such as in DN [23]. In addition, the ablation of the Nrf2 gene has been shown to exacerbate diabetes-induced inflammatory response and aggravate the generation of ROS, thereby inducing DNA oxidative damage and kidney damage [8, 9]. This suggests that Nrf2 is involved in the improvement of diabetes-induced kidney damage. Studies have shown that GSPE can activate the Nrf2 signalling pathway and regulate downstream antioxidant enzymes to combat oxidative stress [12]. In this study, H-GSPE increased the content of Nrf2 in the cell nucleus and reversed decrease in the levels of HO-1 and NQO1, GST activity, and GSH concentration in the downstream target tissues of Nrf2. This suggests that GSPE can partially alleviate oxidative stress by increasing the activity of antioxidant enzymes. Therefore, GSPE plays a key role in the activation of Nrf2, which reduces renal toxicity through antioxidant mechanisms.

It has been reported that higher dietary polyphenol intake can prevent diabetes [24, 25]. Studies have shown that GSPE protects the kidney of STZ-induced diabetic rats by reducing or reversing UAD excretion [19]. GSPE can also protect DN by attenuating endoplasmic reticulum stress-mediated apoptosis through the caspase-12 pathway [17]. These findings show that GSPE is a promising therapeutic agent for DN.

However, there are some limitations in this study. Due to the limitations of time and fundings, we only discussed the protection of GSPE on DN from the Nrf2 signalling pathway, and the subsequent experiments can be supplemented to explore the protective effect of GSPE on DN from the aspects of apoptosis, inflammation, and so on.

## 5. Conclusion

This study through the Nrf2 signalling pathway and oxidative stress to explore the effects of proanthocyanidins on DN demonstrated that GSPE can activate Nrf2 and its downstream antioxidant response genes in the kidney tissues of STZ-induced rats with diabetes and act as a transcription factor for the endogenous antioxidant defence systems, thereby reduce the damage of diabetic renal cells, and slow down the progress of DN. In addition, GSPE can improve STZ-induced hyperglycaemia and reduce insulin resistance in rats, thereby alleviating the ultrastructural changes of the renal tubules. These results suggest that GSPE may be a safe therapeutic option for DN. The potential use of GSPE to prevent hyperglycaemia-induced oxidative stress and its adverse effects will provide the basis for the economic use of new resources in the prevention of diabetes complications.

## Figures and Tables

**Table 1:** Primer sequences for quantitative real-time polymerase chain reaction.

Name	Primer	Sequence	Size (bp)
GAPDH	Forward	5ʹ-ACAGCAACAGGGTGGTGGAC-3ʹ	253
	Reverse	5ʹ-TTTGAGGGTGCAGCGAACTT-3ʹ	

HO-1	Forward	5ʹ-GCATGTCCCAGGATTTGTCC-3ʹ	192
	Reverse	5ʹ-GGTTCTGCTTGTTTCGCTCT-3ʹ	

GST	Forward	5ʹ-ACCTTTTGAGACCCTGCTGT-3ʹ	224
	Reverse	5ʹ-GACGGTTCAAATGGTCAGGG-3ʹ	

Nrf2	Forward	5ʹ-CCCATTGAGGGCTGTGAT-3ʹ	247
	Reverse	5ʹ-TTGGCTGTGCTTTAGGTC -3ʹ	

NQO1	Forward	5ʹ-GATGGGAGGTGGTCGAATCT-3ʹ	197
	Reverse	5ʹ-TATCACCAGGTCTGCAGCTT-3ʹ	

GST: glutathione S-transferase; Nrf2: nuclear factor erythroid 2-related factor 2; NQO1, NAD (P)H quinone oxidoreductase 1.

**Table 2:** Body weight, fasting blood glucose and insulin levels, HOMA-IR, kidney weight, and renal index in control and experimental rats.

	Control	DM	L-GSPE	H-GSPE
Body weight (g)	334.57 ± 19.85	224.43 ± 21.62^*∗*^	258.31 ± 18.81^*∗*,*∗∗*^	264.29 ± 20.44^*∗*,*∗∗*^
FBG (mmol/L)	6.26 ± 0.21	23.80 ± 3.31^*∗*^	17.54 ± 3.77^*∗*,*∗∗*^	16.87 ± 2.93^*∗*,*∗∗*^
Insulin (mU/L)	5.29 ± 0.91	2.73 ± 0.49^*∗*^	3.22 ± 0.59^*∗*,*∗∗*^	3.52 ± 0.55^*∗*,*∗∗*^
HOMA-IR (pmolL)	1.47 ± 0.25	2.90 ± 0.61^*∗*^	1.71 ± 0.39^*∗*,*∗∗*^	1.54 ± 0.21^*∗∗*^
Kidney weight (g)	2.03 ± 0.65	3.17 ± 0.38^*∗*^	2.51 ± 0.45^*∗∗*^	2.45 ± 0.87^*∗∗*^
Renal index	0.63 ± 0.09	1.26 ± 0.28^*∗*^	0.94 ± 0.36^*∗*,*∗∗*^	0.75 ± 0.17^*∗∗*^

Control: normal control group; DM: diabetics group; L-GSPE: the group treated with 125 mg/kg GSPE; H-GSPE: the group treated with 250 mg/kg GSPE; FBG, fasting blood glucose; HOMA-IR, homeostasis model assessment of insulin resistance; GSPE: grape seed proanthocyanidin extract. Values are presented as mean ± SD from 10 rats in each group (^*∗*^*p* < 0.05 vs. the control group; ^*∗∗*^*p* < 0.05 vs. the diabetic group).

## Data Availability

The data for the current study are available from the corresponding author upon reasonable request.
